# Merited Honor

**Published:** 1883-04

**Authors:** 


### Merited Honor.

At the recent commencement exercises of the Medical Department of the University of Louisiana, the degree of Doctor of Medicine was conferred upon Dr. J. G. Fredericks, D.D.S., of New Orleans. Dr. F. has been a prominent member of the dental profession for many years. He is a man of excellent attainments, and superior ability in his profession.

This honor, long ere this, might have been conferred upon him with justice and great propriety.

The Doctor's medical knowledge and experience are much greater and more profound than that of the mere student graduate.

Such recognition of real merit, when judiciously exercised, affords a gratification that is not to be lightly esteemed.

It will fall to Dr. F.'s lot to fill the executive chair of the American Dental Association at its next annual meeting.

Long may he live to enjoy his honors.

